# Mixed chronic scrotal pain secondary to piriformis scarring treated with PRF: case report

**DOI:** 10.3389/fmed.2025.1650218

**Published:** 2025-08-22

**Authors:** Fangfang Ding, Fengqi Fan, Feng Ji, Hua Xu

**Affiliations:** Department of Anesthesiology, Yueyang Hospital of Integrated Traditional Chinese and Western Medicine, Shanghai University of Traditional Chinese Medicine, Shanghai, China

**Keywords:** mixed pain, chronic scrotal pain, chronic pelvic pain syndrome, piriformis muscle scar, pulsed radiofrequency, nerve entrapment

## Abstract

In the complex pathological context of mixed pain, where nociceptive, neuropathic, and nociplastic mechanisms coexist and interact, we present an innovative diagnostic and therapeutic model for refractory chronic scrotal pain (CSP) in a 49-year-old man. The pain originated from pudendal nerve entrapment secondary to piriformis scarring. Comprehensive evaluation revealed mixed pain mechanisms: neuropathic (lancinating pain, S2-S4 dermatomal hypoesthesia, and MRI-confirmed nerve compression), nociceptive (MRI-documented proven inflammation and mechanical stress exacerbation), and nociplastic (central sensitization with prolonged pain duration and psychological comorbidities). To address the stratified intervention needs of mixed pain, we implemented a mechanism-targeted strategy that included ultrasound-guided pulsed radiofrequency (PRF, 42 °C/240 s) at the inferior margin of the piriformis muscle to neuromodulate compressed nerves, perineural ozone/steroid injections to modulate the immunoinflammatory microenvironment, and citalopram to manage central sensitization, with complete pain resolution (VAS 8 → 0) sustained at 3-month follow-up. This case uniquely demonstrates how post-surgical piriformis scarring causes tripartite pain pathogenesis through neuro-immune-myofascial interactions, such as mechanical nerve compression, local inflammation, and subsequent central sensitization. The therapeutic strategy synergistically addressed all three cascades of mechanisms via C-fiber modulation, reduction of pro-inflammatory cytokines, and reversal of neuroplasticity. This study advances the understanding of CSP etiology beyond idiopathic causes by providing a reproducible mechanism-based precision model for mixed pain syndromes and advocating for multidisciplinary management that integrates interventional techniques, pharmacotherapy, and psychorehabilitation.

## 1 Introduction

Chronic pain is not attributable to a single underlying mechanism. Instead, mixed pain, characterized by the complex interplay of multiple pain types, is particularly prevalent among chronic pain conditions (1). Chronic scrotal pain (CSP) is defined as persistent unilateral/bilateral pain in the scrotum lasting for more than 3 months. It affects 0.4–4.7% of men globally and poses a complex diagnostic challenge (2). Despite extensive urological evaluation, 50% of cases remain idiopathic, with over half of the patients reporting persistent pain and reduced quality of life after undergoing conventional therapies (2, 3).

Current understanding attributes CSP primarily to urological pathologies such as infections, varicoceles, and trauma; however, emerging evidence suggests that neuropathic mechanisms involving the pelvic floor may also play a role (1, 2). The innervation of the scrotum by the pudendal, ilioinguinal, and genitofemoral nerves renders it vulnerable to myofascial compression, particularly from the piriformis muscle (4, 5). Notably, 30% of patients with chronic pelvic pain syndrome (CPPS) exhibit concurrent scrotal pain, although pelvic floor contributions remain underrecognized, leading to delayed diagnosis and inadequate treatment of nerve entrapment syndromes (5). Standard diagnostic approaches (such as ultrasound and MRI) identify structural abnormalities but lack precision in localizing nerve compression sites (6). This limitation is particularly significant for extrapelvic conditions such as piriformis scarring. Moreover, postoperative piriformis scarring as a cause of CSP remains undocumented in the literature, and its therapeutic management is unexplored.

We report a novel case of refractory left-sided CSP with mixed pain features in a 49-year-old man, attributed to post-surgical piriformis scarring. This case highlights the first use of ultrasound-guided pulsed radiofrequency (PRF) to target both neuropathic and nociceptive mechanisms, offering a new paradigm for the precision treatment of mixed pain syndromes.

## 2 Case description

Patient consent was obtained. We received a consent-to-disclose form.

A 49-year-old man presented with 18 months of refractory left-sided scrotal pain that was unresponsive to multiple specialist consultations or symptomatic treatments, including hydrocele management. The pain subsequently radiated to the left perineal body and perianal region, characterized by lancinating and tearing pain exacerbated by sitting and sexual activity. His medical history was significant for rectal cancer resection 10 years prior, with full recovery. Physical examination revealed significant pelvic floor hypertonicity with specific tenderness at the 5, 7, and 9 o’clock positions during digital rectal examination, with left piriformis tenderness reproducing pain on FADIR testing, and quantitative sensory testing (CPT) confirmed hypoesthesia in the left perianal region (S2-S4 dermatome). External genitalia and testes were normal. Diagnostic evaluation excluded infection and neoplasms through urinalysis, semen analysis, and Doppler ultrasound. Standardized assessments indicated severe symptoms: Visual Analog Scale (VAS) 8/10, Chronic Prostatitis Symptom Index (CPSI) 32, Generalized Anxiety Disorder-7 (GAD-7) 16, and Hamilton Depression Rating Scale (HAMD) 8.

**Figure 1:**
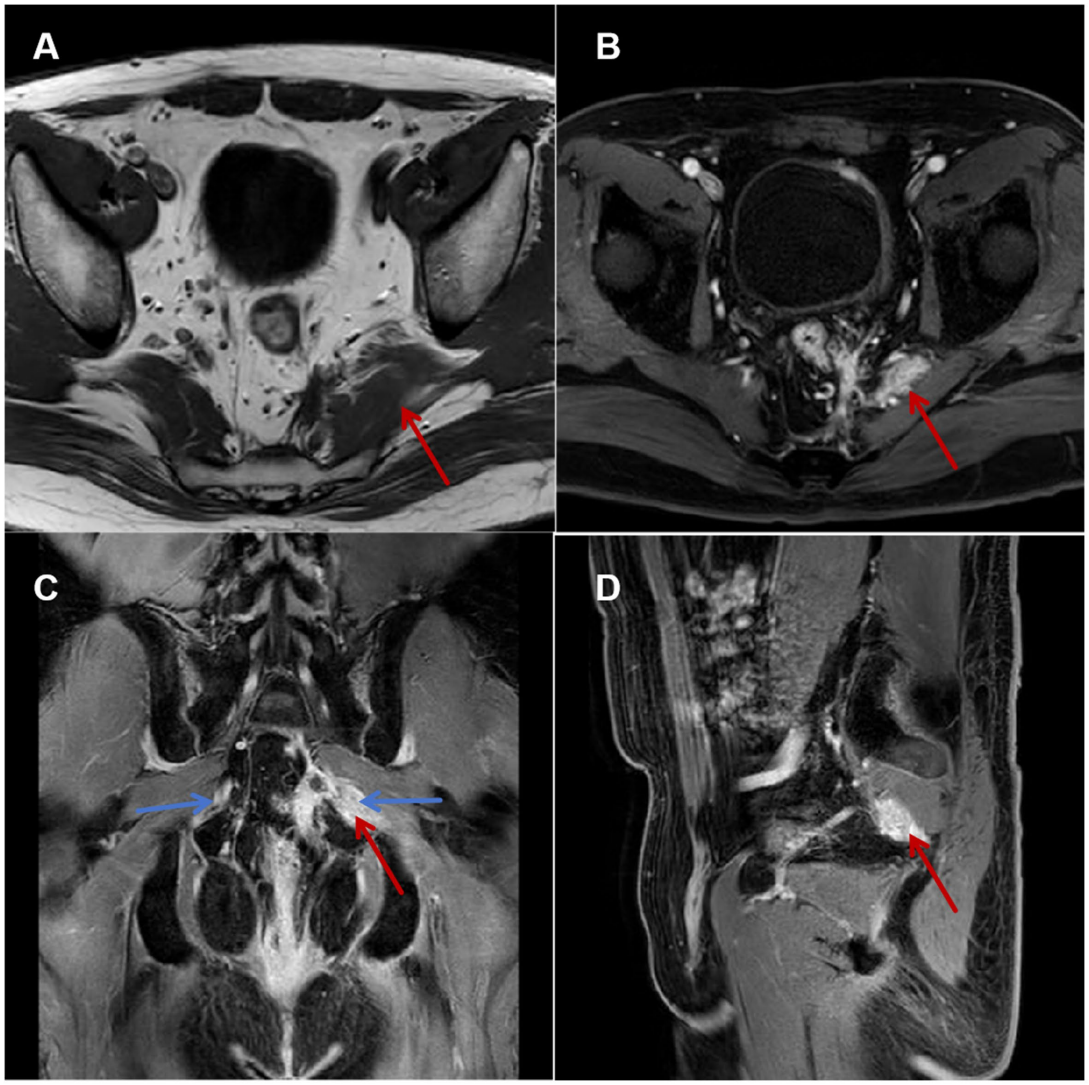
Pelvic floor MRI. **(A)** Axial T1-weighted image: Hypointense signal and thickening of the left piriformis muscle (black arrow). **(B)** Axial T2-weighted image: Heterogeneous hyperintense signal within the left piriformis muscle (black arrow), increased signal intensity in the surrounding fat layer. **(C)** Coronal contrast-enhanced-weighted image: Patchy enhancement surrounding the nerve course (blue arrow) in the left piriformis region, compared to the contralateral side. **(D)** Sagittal contrast-enhanced-weighted image.

Pelvic MRI revealed the following results: (a) T1 hypointensity excluding neoplasms (isointense) or acute hemorrhage (hyperintense); (b) characteristic T2 signal pattern, with hypointensity core (scar fibrosis) surrounded by patchy hyperintensity and ill-defined margins (edema); (c) evidence of perineural adhesion, with T2 hyperintensity in perifascial fat layers surrounding the scar (arrow, Figure 1), and nerve encasement by scar/inflammatory tissue confirmed on contralateral comparison; (d) heterogeneous enhancement confirmed scar-associated inflammation. These findings were independently analyzed and documented by two musculoskeletal radiologists. Based on a comprehensive multimodal evaluation, the patient’s refractory scrotal pain was characterized as a mixed-pain syndrome involving three distinct yet interacting mechanisms. Neuropathic contributions manifested as (a) characteristic tearing pain quality, (b) CPT-confirmed hypoesthesia in the left perianal region, (c) lumbosacral MRI revealed patchy, ill-defined signals within the left piriformis muscle, scar tissue directly abutting the pudendal nerve (Figure 1), and (d) transient of >90% pain relief following diagnostic pudendal nerve block, providing conclusive neurophysiological evidence. Neuropathic pain was confirmed by (a) characteristic tearing pain quality, (b) quantitative sensory testing (CPT) confirming left perianal hypoesthesia, (c) lumbosacral MRI revealing patchy, ill-defined signals within the left piriformis muscle, with scar tissue directly abutting the pudendal nerve (Figure 1), and (d) transient of >90% pain relief following diagnostic pudendal nerve block. Nociplastic mechanisms were identified through (a) pain chronicity (>18 months) exceeding tissue healing timelines, (b) significant psychological comorbidities (GAD-7:16, HAMD:8), and (c) widespread pelvic floor hypertonicity/tenderness beyond the primary scar site, consistent with central sensitization.

Based on the patient’s mixed pain pathophysiology, concomitant pharmacotherapy was initiated on admission: diosmin 0.9 g bid (venoactive), pregabalin 75 mg qn (calcium channel modulator), and mecobalamin 0.5 mg tid (neurotrophic). This regimen was continued for 4 weeks post-PRF to target inflammatory, neuropathic, and reparative mechanisms synergistically.

**Figure 2:**
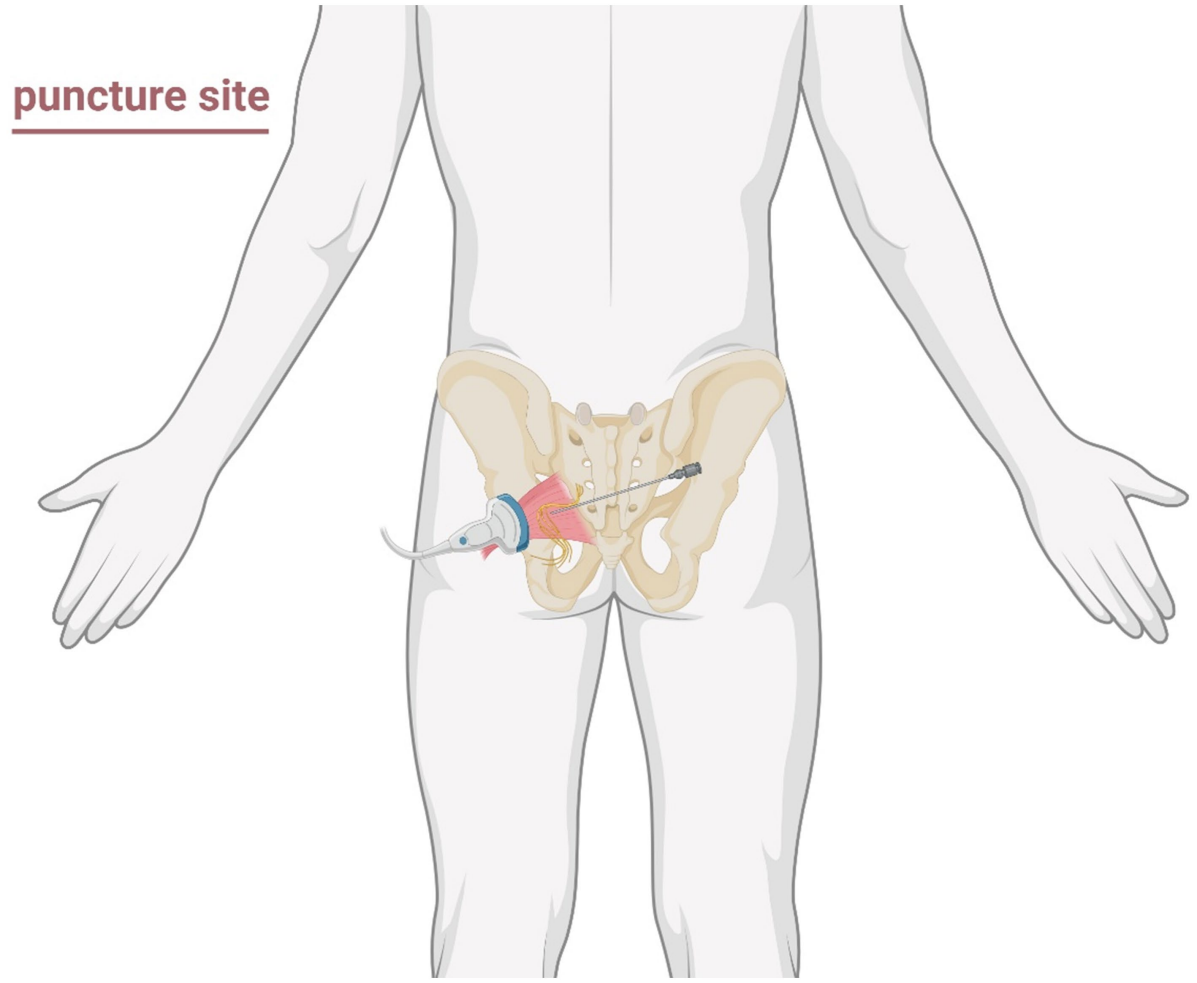
Schematic illustration of the puncture region and approach path for ultrasound-guided pudendal nerve decompression surgery.

**Figure 3:**
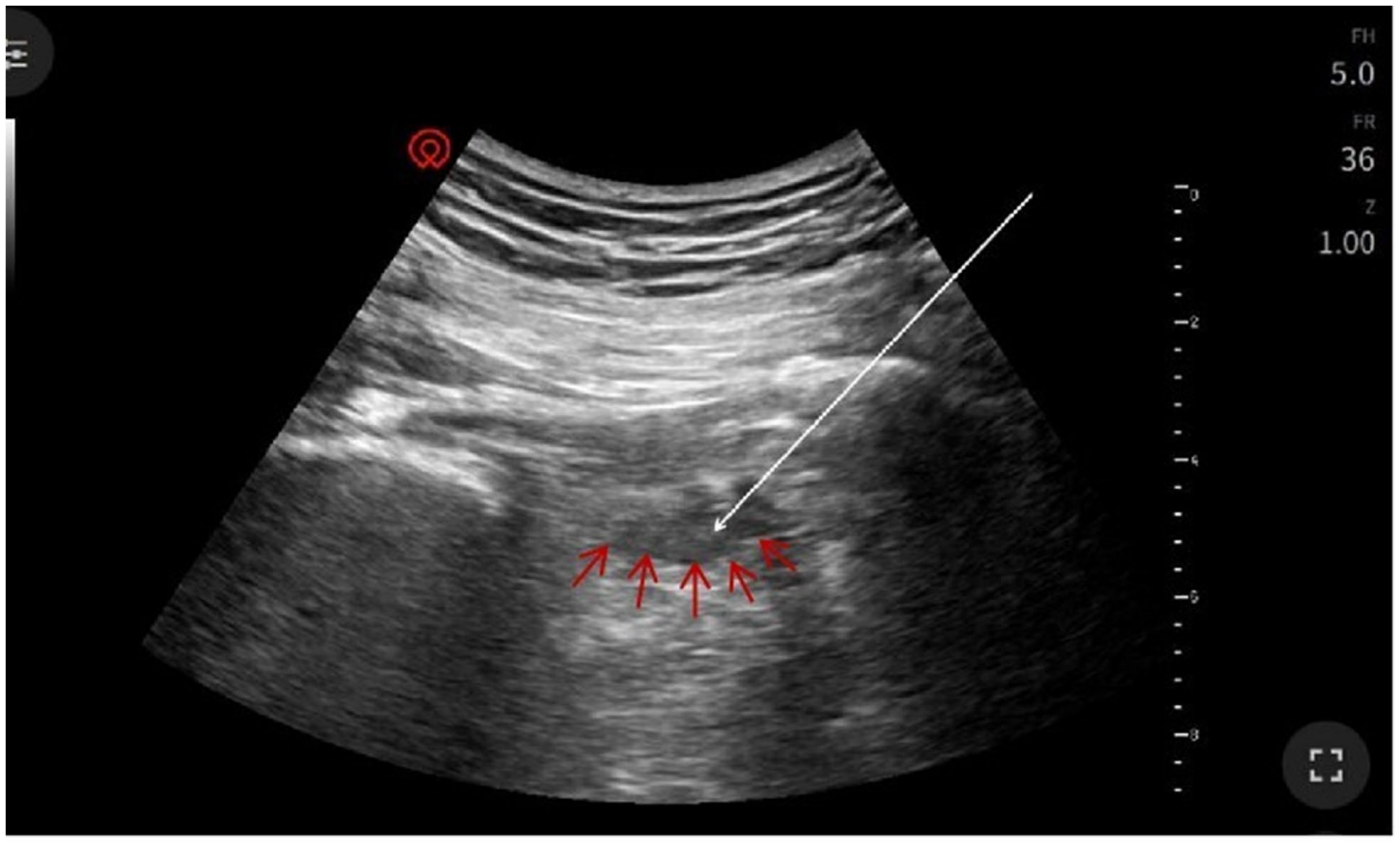
Ultrasound imaging of piriformis muscle: Left piriformis muscle (red arrow) demonstrating surrounding inflammatory signal with enhancement. Needle insertion site (white arrow) indicated at the tip of the arrow.

The patient assumed the prone position with lumbar elevation. Following routine skin disinfection, a 0.7 mm × 80 mm spinal needle (Kindly, China) was inspected for integrity. A color Doppler ultrasound probe (Navis, Wisonic, China; C5-1B micro-convex transducer) was positioned over the medial hip region (Figure 2). Under real-time guidance, the sacrum, greater trochanter, gluteus maximus, and pudendal canal were visualized. The puncture site at the inferior piriformis margin was localized, followed by local infiltration anesthesia of the skin and deep tissues. The needle was advanced in-plane under ultrasound guidance. Hyperechoic regions within the left piriformis muscle indicated fibrotic scarring, perineural adhesions, and nerve compression (Figure 3). Using Doppler guidance, the operator navigated the needle around adjacent vasculature to approach the compressed pudendal nerve. Proximal and distal nerve segments were identified, with 5 mL of normal saline injected at each end to release perineural adhesions. Sensory-motor stimulation elicited paroxysmal pain and muscle contractions, confirming neural targeting. PRF parameters (42 °C, 8 Hz, 30 ms pulse width, 240 s) were applied. A mixture of 0.25% lidocaine 20 mL, mecobalamin 0.5 mg, compound betamethasone 5 mg, and ozone 20 mL (20 μg/mL) was injected perineurally. Post-procedure ultrasound scans (longitudinal and transverse views) confirmed adequate perineural spread. The needle was withdrawn, and the puncture site covered with a sterile dressing. The patient returned to the ward without complications, with intraoperative blood loss recorded as negligible. Postoperative instructions included 2 h of bed rest.

By postoperative day 3, pain had completely resolved (VAS 0/10), with sustained relief at 2-week follow-up accompanied by significant psychological improvement (GAD-75, HAMD 3). At 3 months, the patient reported no recurrence, full resumption of daily activities, and substantial quality-of-life improvement attributed to this minimally invasive approach.

## 3 Discussion

Mixed pain refers to a pain syndrome involving two or more pathological mechanisms simultaneously (7). Common clinical combinations include nociceptive pain (triggered by tissue injury activating nociceptors, e.g., fractures, arthritis) and neuropathic pain (resulting from nervous system damage or dysfunction, e.g., diabetic peripheral neuropathy) (7, 8). Epidemiological data indicate that approximately 30–50% of patients with chronic pain meet the diagnostic criteria for mixed pain, which involves complex mechanisms such as peripheral sensitization, central sensitization, and neuroplastic changes (9–11).

Although chronic pelvic pain syndromes are well-documented, CSP originating from extrapelvic neuromuscular scarring remains exceptionally rare. This report presents the first documented case of piriformis scarring-induced refractory CSP secondary to isolated pudendal nerve entrapment following rectal cancer resection, which was successfully managed with ultrasound-guided PRF neuromodulation. Significantly, this case challenges the conventional paradigm that extrapelvic nerve entrapment must involve sciatic pathology, by demonstrating isolated pudendal neuropathy caused by piriformis scarring at its inferior margin without involving the sciatic nerve, as confirmed by MRI revealing inflammatory adhesion between scar tissue and the pudendal nerve trajectory. We established a triaxial diagnostic framework integrating anatomical mapping (pain distribution/pudendal innervation-MRI scar correlation), electrophysiological verification (CPT-confirmed C-fiber hyperexcitability), and inflammatory imaging (gadolinium-enhanced scar hyperintensity), thereby advancing mixed pain syndrome assessment. Crucially, the initial misdiagnosis as hydrocele-related pain despite normal urogenital ultrasonography underscores a pivotal clinical pitfall. Postoperative pelvic pain requires systematic evaluation of extrapelvic myofascial complexes for fibrotic scarring rather than exclusive genitourinary attribution.

Targeting the inferior piriformis margin (rather than the conventional ischial spine) entails distinct anatomical considerations. The piriformis site lies deeper within the gluteal region, requiring navigation through the gluteus maximus and proximity to the nerve (laterally) and the inferior gluteal neurovascular bundle. To mitigate procedural risks, we implemented three key safety measures: (a) real-time color Doppler ultrasound to map and avoid the inferior gluteal artery/vein during needle advancement; (b) sensory/motor stimulation (0.5–1.0 V, 50 Hz/2 Hz) prior to PRF delivery to confirm proximity to the pudendal nerve (eliciting perineal/genital paresthesia) and absence of motor response in sciatic-innervated muscles (e.g., hamstrings), ensuring the needle tip was not adjacent to the sciatic nerve; (c) hydrodissection with saline prior to injectate/PRF to create a protective fluid buffer around neurovascular structures. While technically more demanding due to depth and proximity of critical structures, ultrasound guidance combined with neurostimulation enabled precise targeting of the scar-nerve interface.

**Figure 4:**
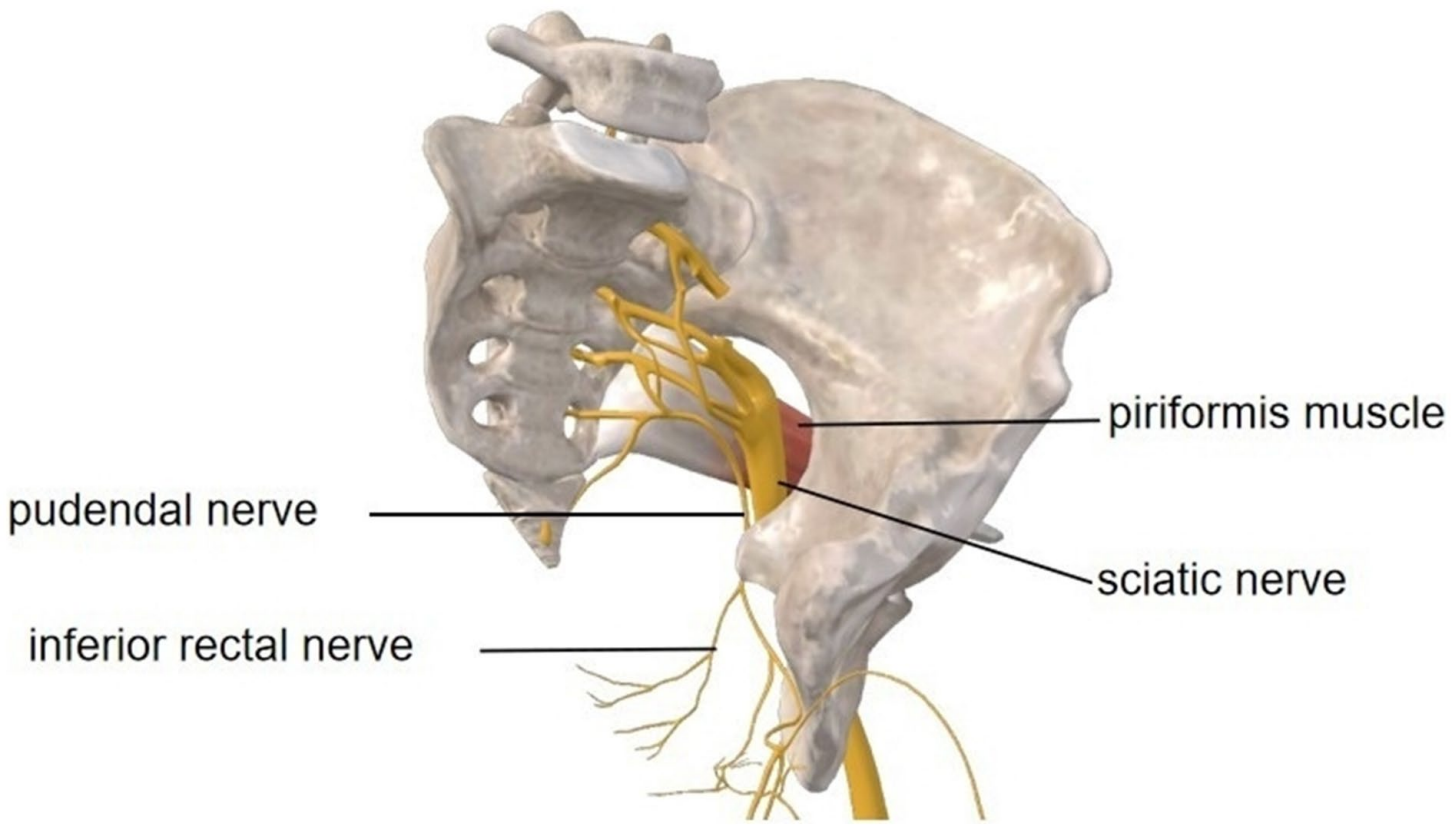
Anatomical pathways of the left pelvic pudendal nerve and sciatic nerve, along with their relationship to the piriformis muscle. Image courtesy of 3D Body (Shanghai, V8.8.51, Copyright obtained from Third Body Digital Technology Co., Ltd.).

Postoperative scarring at the piriformis margin, the superior boundary of the greater sciatic foramen, caused mechanical entrapment of the pudendal nerve (S2-S4), establishing a peripheral sensitization source (Figure 4). This anatomical compression induced neuropathic pain manifesting as lancinating scrotal sensations and perianal hypoesthesia confirmed by CPT testing. Concurrently, chronic scar inflammation, confirmed by MRI hyperintensity and contrast enhancement, perpetuated nociceptive signaling, thus exacerbating mechanical hypersensitivity during activities like sitting or sexual intercourse. Over time, prolonged afferent input dysregulation contributed to nociplastic changes, reflected in elevated anxiety/depression scores and characteristic central sensitization features. Crucially, this case demonstrates that isolated pudendal nerve compression can originate from localized piriformis scarring without sciatic involvement, with the triad of peripheral sensitization, neuroinflammation, and central plasticity forming the pathophysiological axis of this mixed pain syndrome.

Despite significant psychological improvement post-PRF, long-term management remains imperative. This requires scheduled monitoring (VAS, GAD-7/HAMD) at 3/6/12-month intervals, coupled with referral for CBT, pain neuroscience education, and pelvic floor rehabilitation upon recurrence indicators (mood disturbances, functional limitations, or sensitization).

The diagnostic challenges highlighted in this case, particularly the difficulty in localizing nerve compression within complex scar tissue using conventional MRI, underscore an urgent need for advanced imaging analytics. Emerging deep learning approaches, such as analyzing synovial fluid in knee MRI (12) or classification of sperm head morphology (13). Adapting these methods for pelvic neuroimaging could revolutionize mixed-pain management by allowing (a) automated detection of perineural fibrosis/scarring adjacent to nerves like the pudendal, (b) quantification of microstructural nerve changes indicative of entrapment severity, and (c) objective stratification of patients who would benefit most from precision interventions like PRF. Such AI-augmented diagnostics would directly address the localization limitations encountered in our case, where MRI showed inflammatory signals but required expert correlation to infer nerve compression.

Ultrasound-guided PRF addressed this mixed pathophysiology through synergistic mechanisms. Non-thermal neuroregulation using 42 °C pulsed currents selectively modulates C-fiber Nav1.8 channels, reducing ectopic neuronal firing while preserving neural integrity. Simultaneously, perineural hydrodissection with anti-inflammatory agents (lidocaine, mecobalamin, betamethasone/ozone) disrupted adhesions and modulated the pro-inflammatory microenvironment. Precision needle placement, confirmed through real-time sensory/motor stimulation, enabled direct targeting of the primary compression site at the piriformis margin. This technical criticality explains the sustained pain resolution observed in this case, contrasting with transient relief from conventional ischial spine approaches that failed to address the pathoanatomical epicenter. This dual-action strategy exemplifies how emerging neuromodulatory techniques can disrupt the neuropathic-nociceptive-nociplastic axis in refractory mixed pain states. Pharmacotherapy synergized with PRF through multi-target mechanisms. Pregabalin prevented central sensitization rebound while alleviating neuropathic pain and comorbid depression (14). Mecobalamin enhanced axonal regeneration through neuroinflammatory modulation (15). Diosmin reduced perineural edema during hydrodissection while attenuating chronic inflammation and oxidative stress (16, 17). Post-PRF, it played dual roles: mecobalamin supported nerve recovery during Wallerian degeneration, pregabalin mitigated central sensitization resurgence, and diosmin was tapered with clinical normalization. This staged approach, bridging, preventing, and de-escalating, exemplifies rational integration in mixed pain management.

Notably, precision targeting under ultrasound guidance confirmed needle placement via sensory/motor stimulation, allowing safe intervention at the primary compression site (piriformis margin), unlike conventional ischial spine approaches, where symptom recurrence occurred. This technical nuance may explain the durable response where previous blocks failed. In contrast to the findings reported by Kale et al. regarding combined sciatic-pudendal compression requiring surgical intervention (18), our case illustrates that isolated pudendal neuropathy can originate from localized pelvic scarring. This distinction has therapeutic implications: minimally invasive PRF may be sufficient when symptoms align strictly with pudendal territories and imaging confirms focal compression. Nevertheless, given the shared anatomical corridor, clinicians should monitor for sciatic involvement and include pelvic floor rehabilitation in long-term follow-up to detect recurrence related to neural regeneration.

This study is limited by its single-case design and intermediate follow-up duration. Prior evidence indicates that scrotal pain affects 30% of chronic pelvic pain syndrome patients and strongly correlates with pelvic floor tenderness scores (1). The current findings require validation through prospective cohort studies. Future research should consider the following steps: (a) establish long-term efficacy of PRF beyond 6 months; (b) investigate synergistic protocols integrating PRF with pelvic floor rehabilitation; (c) develop deep learning models for diagnostic triaging, including MRI segmentation of pudendal nerve pathways and AI-driven pain phenotyping.

## 4 Conclusion

Ultrasound-guided PRF is an effective, minimally invasive intervention for refractory CSP caused by postsurgical pudendal entrapment, significantly reducing pain, restoring function, and preventing disability progression. Until formal guidelines for mixed pain management are established, a multidisciplinary team (including pain specialists, interventional radiologists, and rehabilitation physicians) should evaluate patients with therapy-resistant pelvic pain for this targeted approach. The occurrence of pudendal neuralgia in patients with a history of pelvic surgery should prompt clinicians to screen for mixed pain risk factors.

## Data Availability

The original contributions presented in the study are included in the article/supplementary material, further inquiries can be directed to the corresponding author.
